# The daily relations between workplace anger, coping strategies, work outcomes, and workplace affiliation

**DOI:** 10.3389/fpsyg.2025.1538914

**Published:** 2025-02-28

**Authors:** Robin Umbra, Ulrike Fasbender

**Affiliations:** Faculty of Business, Economics and Social Sciences, Institute of Education, Work and Society Chair of Business and Organizational Psychology, University of Hohenheim, Stuttgart, Germany

**Keywords:** workplace anger, ruminative and confrontative coping, resource depletion, goal attainment, workplace affiliation disposition

## Abstract

This study examines the daily relations among workplace anger, coping strategies, work outcomes, and employee dispositions using a conceptual framework based on affective events theory and cognitive perspectives on emotions. A sample of 214 full-time employees took part in a two-week study, contributing 1,611 daily observations through an experience sampling approach. Contrary to the assumption that workplace anger always detrimentally relates to work outcomes, the results showed a nonsignificant relation between workplace anger and workplace resource depletion, as well as a positive link between workplace anger and goal achievement. These relations were dependent on the coping strategies used by employees in response to anger-inducing situations, as well as their attitudes toward workplace affiliation. These findings suggest the need to expand affective events theory to include coping strategies as a mediator between affective responses and work outcomes. They also highlight the importance of integrating employee-level factors into organizational research models.

## Introduction

Facilitators of workplace resource depletion, defined as a psychosomatic state characterized by diminished working memory and self-regulation abilities at the workplace (Baumeister et al., 1998), as well as threats to workplace goal attainment, defined as the daily progress toward or achievement of employees’ work-related goals (Sheldon and Elliot, 1999), pose risks to the sustainable functioning of organizations and the societal system (Mohr, 1973; Sekaran and Snodgrass, 1989). Therefore, it is essential for organizations to address factors that could either contribute to or impede these work outcomes (Godkin and Allcorn, 2008; Morrison and Milliken, 2000). One such factor could be workplace anger. Workplace anger refers to an arousing negative emotion experienced when someone has wronged either oneself or those close to oneself (Kant et al., 2013; Lazarus, 1991; Schwarzmüller et al., 2016). It has often been perceived as the opposite of rationality in organizations (Ashkanasy and Dorris, 2017; see also Pham, 2007) and is adversely associated with various work outcomes (Booth et al., 2017; Callister et al., 2017; Gibson et al., 2009). However, it is worth questioning whether the assumption that workplace anger and work outcomes are always adversely related.

Historically, workplace anger has been adversely associated with work outcomes (see Jäger et al., 2017; Wong et al., 2017; but see also Schmitt et al., 2019), a concept that is supported by organizational behavior theories. Affective events theory (Weiss and Cropanzano, 1996), for instance, suggests that negative emotions, such as anger, can facilitate disadvantageous and impede advantageous work outcomes. This theoretical perspective is further supported by empirical research, which consistently shows an adverse relation between negative emotions and work outcomes (Wong et al., 2017). Nevertheless, it is important to consider potential limitations in theoretical frameworks and empirical studies, which may warrant further exploration.

In exploring the limitations of current conceptual models in understanding the relation between workplace anger and work outcomes, it is evident that a significant gap exists in the lack of consideration of anger’s coping strategies, such as ruminative (excessive internal pondering about an anger-inducing situation; Li et al., 2019) and confrontative coping (openly and antagonistically addressing an anger-inducing situation; Folkman et al., 1986). While affective events theory and empirical research have laid valuable foundations for the organizational sciences, they seem to often miss to address the role of these and other coping strategies in managing emotions in the workplace (e.g., Carlson et al., 2011; Glasø et al., 2010; Wegge et al., 2006). This oversight becomes particularly striking when compared to alternative frameworks, such as cognitivist accounts of emotion (Lazarus, 1991; Frijda, 1987; Moors et al., 2013), which highlight the importance of coping mechanisms in daily working life. It would thus seem reasonable to assume that how individuals cope with anger at work could intersect the relation between their anger and their work outcomes. However, further research is needed to validate this assumption and determine if coping strategies indeed play a substantial role in the connection between workplace anger and work outcomes. Furthermore, while existing research has focused on the influence of organization-and supervisor-level factors on the relation between emotions and work outcomes, there is a noticeable gap in the empirical literature when it comes to examining employee-level constructs, such as dispositions (cf. Glasø et al., 2010; Wegge et al., 2006), within the framework of affective events theory (Weiss and Cropanzano, 1996). This lack of research is significant because alternative theoretical models rooted in cognitivist accounts of emotions also highlight the importance of individual differences in understanding emotions in the workplace. As such, a key question arises regarding whether the relation between workplace anger and work outcomes is consistent for all employees or indeed varies based on their unique dispositions. For instance, an individual’s workplace affiliation disposition, which pertains to their inherent desire to be part of a team or group at work (Yagil and Medler-Liraz, 2017), could play a critical role in how they cope with workplace anger and, subsequently, relate to their work outcomes. In light of this, it is crucial to delve deeper into such employee-level factors within appraisal theory to gain a comprehensive understanding of how it impacts the relations between workplace anger and work outcomes.

As such, our primary objective in this manuscript is to examine the intricate relations between workplace anger and work outcomes, taking into account the potential role of coping strategies and individual differences. To achieve this objective, we have devised a conceptual model based on affective events theory (Weiss and Cropanzano, 1996) and enhancements from cognitivist accounts of emotion (Lazarus, 1991; Frijda, 1987; Moors et al., 2013). Our conceptual model assumes that workplace anger can be disadvantageously associated with work outcomes, but also considers the possibility that this relation may be advantageous under certain circumstances. These circumstances include the coping strategies employed to address workplace anger and the individual differences that may exist among employees. To test our conceptual model, we will gather data from a sample of full-time employees across various industries over a two-week period. Data will be collected each workday using a time-lagged experience-sampling methodology.

Through our current work, we aim to make a significant contribution to the existing literature in three key areas. Firstly, we seek to investigate the assumption of affective events theory (Weiss and Cropanzano, 1996) and cognitivist accounts of emotion (Lazarus, 1991; Frijda, 1987; Moors et al., 2013) regarding the relation between workplace anger and work outcomes, such as work-related resource depletion. While both theories suggest a negative link between workplace anger and resource depletion, empirical evidence supporting this claim is still lacking. By delving into affective events theory and cognitivist accounts of emotions, we aim to clarify the true nature of this relation in work environments. Therefore, our objective is to test the validity of these theories in explaining the connections between workplace anger and resource depletion.

Moreover, we aim to make a theoretical contribution by examining the differing assumptions about the relation between workplace anger and goal attainment in affective events theory (Weiss and Cropanzano, 1996) and cognitivist accounts of emotions (Lazarus, 1991; Frijda, 1987; Moors et al., 2013). We suggest that the discrepancy in these assumptions may stem from the lack of emphasis on coping strategies in affective events theory compared to cognitivist accounts of emotions, which prioritize coping strategies. Our hypothesis is that coping strategies associated with workplace anger can significantly influence the relation between workplace anger and goal attainment. By incorporating coping strategies such as ruminative coping (excessive internal pondering about an anger-inducing situation; Li et al., 2019) and confrontative coping (openly and antagonistically addressing an anger-inducing situation; Folkman et al., 1986) into our conceptual model, we aim to explore how these strategies may impact the relation between workplace anger and goal attainment.

Finally, our study aims to examine the impact of individual differences on the relation between workplace anger and work outcomes. Previous research has overlooked employee-level factors within the context of affective events theory. To address this gap, we include the core human disposition of workplace affiliation in our conceptual model (Ryan and Vansteenkiste, 2023). This disposition is in line with the interaction-oriented theoretical frameworks of affective events theory and cognitivist accounts, suggesting that it may influence the interconnectedness of workplace anger, coping strategies, and work outcomes. By investigating employee-level factors through the lens of affective events theory, we strive to gain a better understanding of the importance of individual differences within this theoretical framework.

## Theoretical background and hypotheses development

### Affective events theory

Affective events theory is an affect-centered theory from the organizational sciences that focuses on the relation between emotions arising from workplace situations and the work outcomes that are expected to result from these emotions (Weiss and Cropanzano, 1996). According to the theory, emotions and work outcomes typically have similar valences. Positive emotions are likely to lead to positive work outcomes, while negative emotions are likely to lead to negative work outcomes. The theory also suggests that employee-level factors, such as dispositions, are important in understanding this relation. However, the specific impact of dispositional factors on the relation between emotions and work outcomes has not been extensively researched and remains a theoretical assumption at this time. It is worth noting that affective events theory does not expressively address the role of coping mechanisms within its theoretical framework.

#### Cognitivist accounts of emotions and intertheoretical discourse

Cognitivist accounts of emotions challenge the notion of affective events theory that emotions and work outcomes always align in terms of valence. Cognitivist accounts propose that an employee’s coping strategies mediate the valence of an emotion onto the valence of a workplace outcome (Frijda, 1987; Lazarus, 1991; Moors et al., 2013). Two main coping strategies typically available to employees in such situations are emotion-focused coping and problem-focused coping (Lazarus, 1991).

Emotion-focused coping involves managing one’s emotions through internal thought regulation processes, while problem-focused coping entails actively addressing the situation itself (Lazarus, 1991). Cognitivist accounts of emotions suggest that regardless of the initial emotion triggered by the situation, emotion-focused coping tends to have a negative mediating effect on the relation between emotions and work outcomes (Folkman et al., 1986; Lazarus, 1991; Roseman, 2013). In contrast, problem-focused coping is believed to have a positive mediating effect on this relation. Empirical evidence seems to support these assumptions regarding the mediating role of coping strategies in the relation between emotions and work outcomes (Baker and Berenbaum, 2007; Boyd et al., 2009; Carver, 2006).

Cognitivist accounts of emotion align with affective events theory in recognizing the significance of individual differences (Lazarus, 1991; Frijda, 1987; Moors et al., 2013), such as dispositions, in the link between emotions and work outcomes. However, there is a difference in perspective between the two theories. While affective events theory suggests that dispositions directly impact the relation between emotions and work outcomes (Weiss and Cropanzano, 1996), cognitivist accounts propose that dispositions impact this relation indirectly (Lazarus, 1991; Frijda, 1987; Moors et al., 2013).

According to cognitivist accounts of emotion, dispositions affect the mediation path between emotions and coping strategies of employees, as well as the mediation path between coping strategies and work outcomes (Lazarus, 1991; Frijda, 1987; Moors et al., 2013). In contrast, affective events theory suggests that individual differences impact the direct relation between emotions and work outcomes (Weiss and Cropanzano, 1996). This means that in cognitivist accounts, the connections between emotions and coping strategies, as well as coping strategies and work outcomes, are moderated by dispositions. On the other hand, affective events theory maintains that only the direct link between emotions and work outcomes is moderated by dispositions (Weiss and Cropanzano, 1996).

**Figure 1:**
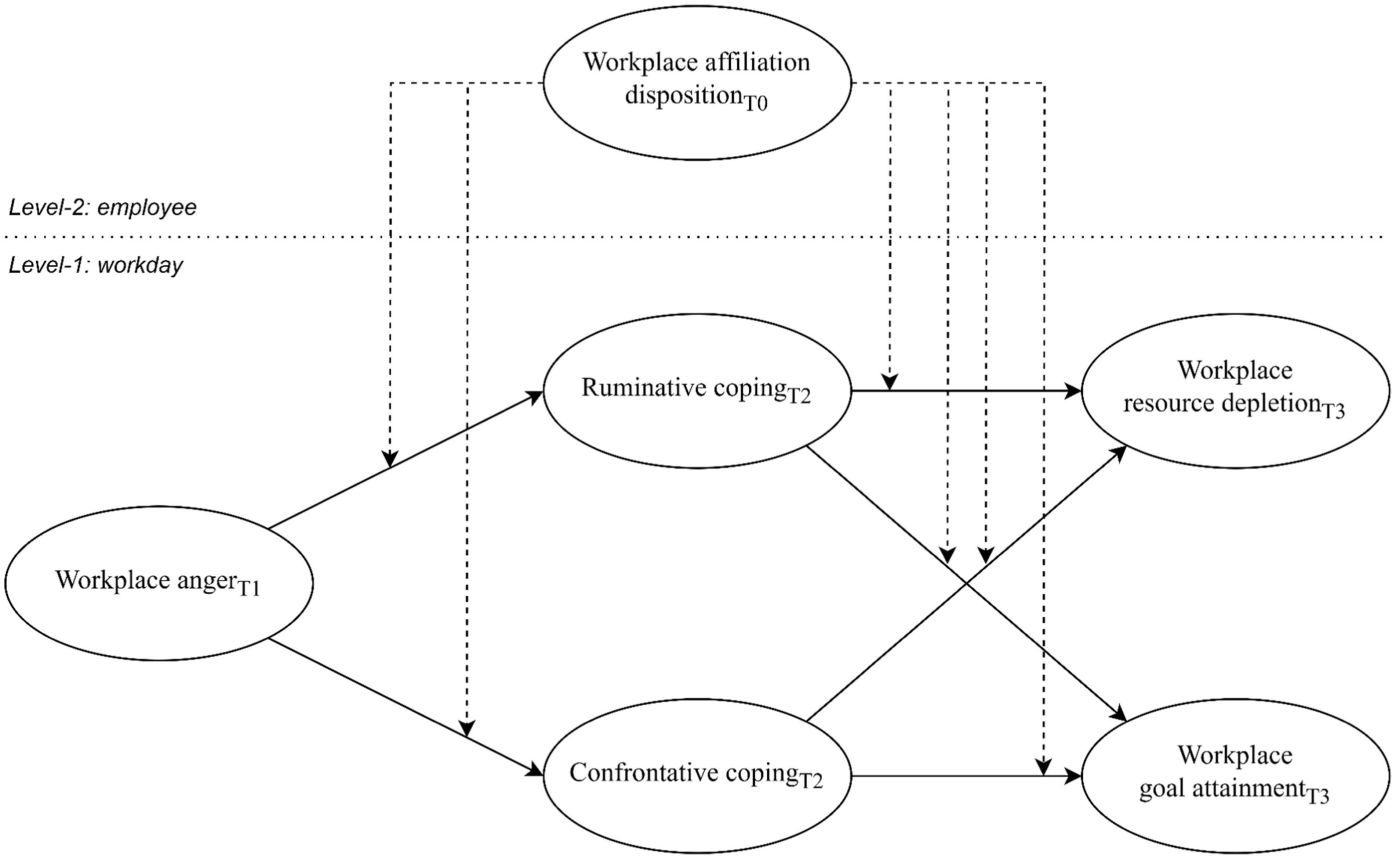
Conceptual model.

In summary, affective events theory and cognitivist accounts of emotion offer different theoretical assumptions when it comes to the relation between emotions and work outcomes. Affective events theory suggests a same-valenced and direct link between emotions and work outcomes, with employee dispositions playing a significant role in shaping this direct relation (Weiss and Cropanzano, 1996). On the other hand, cognitivist accounts of emotion propose that coping strategies play a mediator role in concluding the relation between emotions and work outcomes, with employee dispositions presumably also impacting this relation (Lazarus, 1991; Frijda, 1987; Moors et al., 2013). We will now delve deeper into these discrepancies and their possible resolutions, following the procedural structure of our conceptual model depicted in Figure 1.

#### A case for workplace anger

In light of the conflicting perspectives presented by different theoretical frameworks, it is crucial to establish which framework better aligns with evidence regarding their assumptions. One emotion that holds significance in workplace settings and may provide insight in this regard is anger. Anger (an arousing negative emotion that is experienced when someone has wronged either oneself or those close to oneself; Lazarus, 1991) seems to be a particularly fitting emotion for our investigation because it is commonly viewed as a negative emotion (Lazarus, 1991; Weiss and Cropanzano, 1996), suggesting, according to affective events theory (Weiss and Cropanzano, 1996), that it should have a detrimental impact on work outcomes. However, from a cognitivist perspective (Lazarus, 1991; Frijda, 1987; Moors et al., 2013), anger is also seen as an adaptive emotion due to its coping strategies, implying a potentially positive relation with work outcomes. Therefore, in this manuscript, our focus will be on exploring the relation between anger in the workplace (referred to as workplace anger) and work outcomes.

#### The cases for workplace resource depletion and workplace goal attainment

Building on the argument above, it is important to identify specific work outcomes that we can use to test our two theoretical frameworks. Workplace resource depletion, a psychosomatic state characterized by diminished working memory and self-regulation abilities at the workplace (Baumeister et al., 1998), and workplace goal attainment, the progress toward or achievement of work-related goals (Sheldon and Elliot, 1999), seem to stand out as suitable work outcomes for our analysis. Not only are they crucially important for organizations (Kiresuk et al., 2014; Mohr, 1973; Sekaran and Snodgrass, 1989), but they also highlight a significant discrepancy between our two theoretical perspectives in terms of their relation to workplace anger.

According to affective events theory (Weiss and Cropanzano, 1996), workplace resource depletion, as a negative work outcome, should be positively related to anger, which is considered a negative emotion. Cognitivist perspectives of emotion seem to support this view, as all emotions and coping strategies must, by their nature, be related to higher resource depletion (Lazarus, 1991; Vohs and Baumeister, 2011; Vohs and Heatherton, 2000).

However, this intertheoretical alignment disappears when we consider workplace goal attainment. This positive work outcome should be inversely related to anger according to affective events theory (Baumeister et al., 1998; Weiss and Cropanzano, 1996). In contrast, cognitivist perspectives on emotion (Lazarus, 1991; Frijda, 1987; Moors et al., 2013) suggest that the relation between workplace anger and goal attainment could be either negative or positive, depending on the employee’s coping strategies.

Given these intertheoretical alignments and discrepancies, our study will focus on exploring the connections between workplace anger and workplace resource depletion and goal attainment, due to its theoretical and practical relevance. With this focus in mind, we will now delve into the reasons why such intertheoretical discrepancies might exist and propose our hypotheses accordingly.

#### The relations between workplace anger and coping strategies

We have established that the anticipated results may differ when viewed through competing theoretical frameworks. Specifically, we argue that the variation in these results can be attributed to the differing emphasis placed on coping strategies in affective events theory (Weiss and Cropanzano, 1996) compared to cognitivist theories of emotions (Frijda, 1987; Lazarus, 1991; Moors et al., 2013). While cognitivist theories highlight coping strategies as crucial elements, affective events theory tends to downplay their importance (Weiss and Cropanzano, 1996). Therefore, our assumption suggests that coping strategies associated with workplace anger may significantly impact the direction of the relation between workplace anger and workplace resource depletion, as well as goal attainment. In essence, coping strategies could determine whether this connection is positive or negative. To further explore this hypothesis, we will examine the potential role of coping strategies in shaping the link between workplace anger and workplace resource depletion, as well as goal attainment.

According to cognitivist theories, employees can employ either emotion-focused coping or problem-focused coping strategies to handle situations that trigger emotions in the workplace (Lazarus, 1991). Emotion-focused coping involves internally regulating emotions, while problem-focused coping involves taking action to address the situation that caused the emotion. Emotion-focused coping strategies related to anger often involve excessive internal rumination about the anger-inducing situation, known as ruminative coping (Folkman et al., 1986; Folkman and Lazarus, 1985; Lazarus, 1991). On the other hand, problem-focused coping strategies associated with anger typically involve directly confronting the anger-inducing situation, referred to as confrontative coping. Based on this, we propose the following two hypotheses to be tested:

Hypothesis 1: There is a positive relation between workplace anger and (a) ruminative coping as well as (b) confrontative coping.

#### The relations between coping strategies and work outcomes

The relation between coping strategies, specifically ruminative and confrontative coping, and workplace resource depletion is well-established, as there is consensus across various theories that any type of regulation, whether directed toward others or oneself, depletes internal resources (Baumeister et al., 1998; Lazarus, 1991; Weiss and Cropanzano, 1996). Therefore, we hypothesize that both primary anger coping strategies, ruminative and confrontative coping, are positively related with workplace resource depletion. Though initially grounded in theory, empirical evidence supports the idea that these relations are as expected (see Keltikangas-Järvinen et al., 1996; Vohs and Heatherton, 2000). Therefore, we suggest testing the following hypotheses:

Hypothesis 2: There is a (a) positive relation between ruminative coping and workplace resource depletion as well as a (b) positive relation between confrontative coping and workplace resource depletion.

However, as mentioned previously, this theoretical alignment breaks down when examining the relation between coping strategies and workplace goal achievement. Specifically, cognitivist views suggest that, in general, using emotion-focused coping may impede goal achievement, while utilizing a problem-focused coping strategy can be advantageous to achieve goals (see Folkman et al., 1986; Lazarus, 1991; Roseman, 2013). This notion is supported by empirical evidence (Baker and Berenbaum, 2007; Boyd et al., 2009; Carver, 2006).

In our assertion, we propose that the relation between coping strategies and workplace goal attainment should follow a similar pattern. Therefore, it is crucial to explore the coping strategies associated with anger in the workplace. With this understanding, it is reasonable to hypothesize that ruminative coping may negatively relate to workplace goal attainment, while confrontative coping may have a positive relation.

The dichotomy between emotion-focused coping and confrontative coping lies in their abilities to change the anger-inducing situation (Lazarus, 1991; Lazarus and Folkman, 1984). Emotion-focused coping may change how an employee subjectively interprets a situation, but it does not objectively change the situation itself. In contrast, confrontative coping has the potential to directly change the anger-inducing situation. Unresolved anger-inducing situations can lead to employees expending cognitive resources on managing their emotions rather than focusing on workplace goals (see also Baumeister and Vohs, 2004; Vohs and Heatherton, 2000). Problem-focused coping that addresses or resolves the anger-inducing situation can free up cognitive resources for employees to focus on their goals.

Empirical evidence suggests that ruminative self-focus, which could be regarded as a characteristic of ruminative coping (see Frijda, 1987), is negatively related to goal attainment (Moberly and Watkins, 2009), while approach behavior, a component of confrontative coping (Lazarus, 1991), is positively associated with goal attainment (Brodscholl et al., 2007). As such, along with our theoretical rationale, we propose the following hypotheses:

Hypothesis 3: There is a (a) negative relation between ruminative coping and workplace goal attainment as well as a (b) positive relation between confrontative coping and workplace goal attainment.

#### The indirect relations between workplace anger and work outcomes

So far, we have examined the assumed direct relations between our theoretical constructs. However, it is important to note that both affective events theory and cognitive accounts of emotions suggest a procedural nature of these relations and constructs. As our main focus in this paper is to determine if the relation between workplace anger and work outcomes is consistently positive when taking coping strategies into account, it is important to investigate this in a more detailed manner. Therefore, we plan to explore the indirect effects between workplace anger and work outcomes through the use of coping strategies. Building upon the hypotheses we have developed regarding their direct effects, we propose four mediation hypotheses to be tested in our research:

Hypothesis 4: There is a (a) positive relation between workplace anger and workplace resource depletion via ruminative coping as well as a (b) positive relation between workplace anger and workplace resource depletion via confrontative coping.

Hypothesis 5: There is a (a) negative relation between workplace anger and workplace goal attainment via ruminative coping as well as a (b) positive relation between workplace anger and workplace goal attainment via confrontative coping.

#### A case for workplace affiliation dispositions

As we compare the core assumptions of different theoretical frameworks and their alignment with empirical evidence, it is important to also consider the validity of their auxiliary assumptions. One key aspect to consider is the moderating impact of employee dispositions on the assumed relations. This is especially crucial as it illuminates another significant theoretical discrepancy between affective events theory and cognitivist accounts of emotion (Lazarus, 1991; Frijda, 1987; Moors et al., 2013). Essentially, in cognitivist accounts, the relations between emotions and coping strategies, and between coping strategies and work outcomes, are moderated by employee dispositions. In contrast, affective events theory argues that only the direct link between emotions and work outcomes is moderated by employee dispositions (Weiss and Cropanzano, 1996).

To assert the incremental validity of the two theoretical frameworks, we may want to consider examining a distinct disposition to test our theoretical assumptions with. Workplace affiliation (an inherent desire to be part of a team or group at work; Yagil and Medler-Liraz, 2017) seems to be a promising choice for this analysis as it seems to be regarded as a disposition in both frameworks (Lazarus, 1991; Weiss and Cropanzano, 1996), has a strong association with anger due to anger’s contrary affiliative nature (Lazarus, 1991; Weiss and Cropanzano, 1996; see also Oatley, 2009), and is a distinct and measurable (Yagil and Medler-Liraz, 2017) construct. Thus, our research will concentrate on investigating the role of workplace affiliation in moderating the connections between workplace anger, coping strategies, and workplace resource depletion as well as goal attainment. This leads us to formulate and subsequently investigate the following research question:

Research question: How does an employee’s workplace affiliation disposition impact the relations between workplace anger, coping strategies, and workplace resource depletion as well as goal attainment?

### Methodological congruence

In order to address the challenges of methodological incongruence in the organizational sciences (Thurston et al., 2008), our research aims to align our methodology closely with the nature of our theoretical constructs to minimize any potential misfit that may arise. One key consideration in this alignment is the temporal volatility of our constructs. Constructs such as workplace anger, workplace resource depletion and goal attainment, and coping strategies like ruminative and confrontative coping are likely to fluctuate daily (Lazarus, 1991; Weiss and Cropanzano, 1996), while a workplace affiliation disposition tends to change less rapidly. Furthermore, considering our theoretical frameworks that suggest a procedural relation between workplace anger, coping strategies, and workplace resource depletion as well as goal attainment (Lazarus, 1991; Weiss and Cropanzano, 1996), it is also vital to select a methodological approach that can capture this sequential nature.

We identified a daily time-lagged experience-sampling approach as a suitable option, as it can accommodate the differing volatilities of our constructs and the procedural relations we assume (Gabriel et al., 2019; Ohly et al., 2010; Trull and Ebner-Priemer, 2009). While this approach offers the advantage of increased external validity (Findley et al., 2021), concerns about internal validity and causality may arise. However, given the partly exploratory nature of our research, we consider that using an experience-sampling approach would provide the most significant theoretical and practical insights.

## Method

### Transparency and openness

We preregistered our hypotheses, study, and analysis plan on the Open Science Framework, accessible at https://osf.io/3tdwv/?view_only=30658bc72bc743e28775dd6876cce1d5. The data summary and analysis code will also be available in the same directory upon publication. Informed consent was acquired from every participant prior to data collection.

### Selection and procedure

The research conducted in this study was ethically approved by the committee at authors’ university. Our goal was to obtain a diverse sample of employed adults who worked a minimum of 30 h per week, with the exclusion of employees under 18 or over 67 years old. By recruiting participants through student networks, we utilized a comparable and conventionally robust approach to Burmeister et al. (2020) and Fasbender et al. (2021), thereby gaining a wide variety of participants from different networks. To capture changes in participants’ experiences, we utilized an experience sampling format over 10 consecutive workdays (Gabriel et al., 2019). As an incentive for participation, participants received gift vouchers. Surveys were sent to participants at three specific times daily: 9:30 a.m. (morning survey; at work), 12:30 p.m. (noon survey; at work), and 3:30 p.m. (evening survey; at work). Data collection occurred between May and December 2022. Our study’s attrition rate was consistent with previous research (Ohly et al., 2010; Xia et al., 2021), with a 9% dropout rate and a 68% compliance rate for daily surveys. We observed no significant effects of gender or age on attrition rates.

### Sample characteristics

A total of 1,611 observations were collected on the employee-day experiences of 214 participants. The average number of observations per employee was 7.53. This sample size exceeds the standard experience sampling standards outlined by Gabriel et al. (2019). Of the participants, 60% were female, with an average age of 34.83 years (SD = 13.08) and a weekly work schedule of 39.26 h (SD = 4.80). Their average general work experience was 15.08 years (SD = 12.95), and they had been with their organization for an average of 9.23 years (SD = 9.87). Of the participants, 26% held leadership positions, while 74% performed office duties. Additionally, 26% performed non-office tasks, and 66% worked in an office environment. Furthermore, 13% worked from home, 11% at customers’, and 10% in public locations. Regarding company size, 58% worked in large companies, 16% in medium-sized firms, 15% in small businesses, and 11% in micro-companies. Lastly, 17% worked in banking, finance, and insurance, 16% in production/manufacturing, 10% in public administration, 9% in healthcare, 7% in IT and communications, 6% in craft trades, 6% in education and training, 5% in retail/wholesale trade, 3% in energy and water supply, 2% in catering/hospitality, 2% in transportation, less than 1% in agriculture and forestry, less than 1% in science, and 16% in other industries.

### Measures

The translation of the measurement scales from English to German was conducted using the back-translation method described by Brislin (1970). Unless otherwise stated, a 5-point Likert scale was utilized, with response options ranging from 1 (indicating strong disagreement) to 5 (indicating strong agreement). Our selection of items was determined by considering factor loading matrices and theoretical congruence while prioritizing the minimization of participant burden by keeping the number of items low (Gabriel et al., 2019). We adapted the scale to the workplace and the study context by adding “Today at work, …” to each item.

#### Workplace anger (morning survey)

To assess workplace anger, we utilized four items from Spielberger et al.’s (1983) scale, whereby participants were asked to indicate their present experience of anger. An example statement was, “Right now, I feel angry.” The internal consistency of this scale was high, with a McDonald’s Omega (*ω*; McDonald, 1999; see also Geldhof et al., 2014) value of 0.95.

#### Ruminative coping (noon survey)

Ruminative coping was evaluated using the 3-item scale developed by Li et al. (2019). An example of a sample item was, “Throughout my workday today, I could not stop thinking about an event that made me angry.” The scale exhibited a high level of reliability (*ω* = 0.95).

#### Confrontative coping (noon survey)

We evaluated confrontative coping utilizing a scale developed by Folkman et al. (1986). A sample item is, “Today at work, since the beginning of my workday, I stood my ground and fought for what I wanted” (*ω* = 0.76).

#### Workplace resource depletion (evening survey)

We employed a set of three items derived from Lanaj et al.’s (2014) scale to monitor workplace resource depletion. One of the sample questions included in this set was, “Today at work, since filling out the last questionnaire, my mind has felt unfocused” (*ω* = 0.87).

#### Workplace goal attainment (evening survey)

To guarantee the precision of measuring workplace goal attainment, we have integrated the two items from Judge et al.’s (2005) goal attainment scale and added one item (“Today at work, since filling out the last questionnaire, I achieved my goals at work”), ensuring a minimum of three items. The scale had an omega value of *ω* = 0.91.

#### Workplace affiliation disposition (baseline survey)

In the baseline survey, we utilized the 4-item scale developed by Van Yperen et al. (2014) to measure the participant’s workplace affiliation disposition. An example item from the scale reads, “I am a person who feels the need to feel like they are part of a team or group at work.” (*ω* = 0.86).

#### Controls

Drawing on our theoretical framework, we posit that the experience of negative affect in the morning, such as sadness or anxiety, may impact how employees employ coping strategies later in the day (Lazarus, 1991; Weiss and Cropanzano, 1996). To gauge these morning affects, we utilized a scale deployed by Sonnentag et al. (2008, see also Watson et al., 1988) that assessed negative affect, achieving a solid internal consistency with a McDonald’s omega of 0.92. We determined that our results remained consistent regardless of whether we included or excluded controls, with one exception. The moderation effect of workplace affiliation dispositions on the relation between confrontative coping and goal attainment was found to be borderline non-significant (E(*moderation effect*) = 0.15, 95% CI [−0.001, 0.330], *p* = 0.05). In this connection, we will be reporting the results of our controlled effects moving forward.

### Analytical strategy and data diagnostics

In our study, we conducted data preparation using R version 4.2.2 (R Core Team, 2022) and data analysis using Mplus version 8.4 (Muthén and Muthén, 2017). The dataset consisted of multiple observations for each employee (Hayes, 2006), prompting us to utilize 2-level multilevel modeling with random intercepts and slopes (Hamaker and Muthén, 2020). Due to the presence of non-normality, model complexity, and outliers, we employed Bayesian inference in our analysis, which has conventionally been robust to these issues (Asparouhov and Muthén, 2021; Depaoli, 2021).

We utilized the Gibbs sampler algorithm with a 1.02 Gelman-Rubin potential scale reduction factor (Gelman et al., 2013) and ran two Markov chains for 50,700 iterations. To ensure convergence, we visually inspected trace and autocorrelation plots. Our model estimates were presented using the median as a point estimate, with diffuse priors^1^ used for parameter interpretation similar to traditional maximum likelihood estimation (cf. Depaoli, 2021). Missing data were included in our model estimation (Finch and Bolin, 2017).

Group-mean centering of the predictor variable was automatically applied through confirmatory factor analysis (see Muthén and Muthén, 2017). Outliers were retained in our analysis (Grubbs, 1950; see also Asparouhov and Muthén, 2021; Finch and Bolin, 2017). For level-1 observations, only participants who worked during specific sampling time intervals, as measured in each questionnaire, were included. We included the direct effect of workplace anger on workplace resource depletion and goal attainment, which adhered to the guidelines provided by Kline (2015).

Data diagnostics revealed no abnormalities in data quality (Cullen and Frey, 1999; Delignette-Muller and Dutang, 2015; Gabriel et al., 2019). The mean percentage of missing responses across variables was 23.68%, with specific rates of 16.60% for workplace anger, 25.60% for ruminative and confrontative coping, and 26.90% for workplace goal attainment and resource depletion.

## Results

### Preliminary analyses

The descriptive statistics for the focal variables are presented in Table 1. Results indicate that the participants exhibited low levels of workplace anger, ruminative coping, confrontative coping, and workplace resource depletion while presenting moderate levels of workplace goal attainment. Notably, all variables displayed significant variance on both levels, with a near-equal spread.

**Table 1:** Means, standard deviations, interclass correlation coefficients, reliabilities, and correlations among the focal factors.

Variables	*M*	*SD*_Level-2_	*SD*_Level-1_	ICC	1.	2.	3.	4.	5.	6.
1. Workplace anger_T1_	1.42	0.56	0.63	0.47	(0.95)	0.80*	0.77*	0.57*	−0.39*	0.01
2. Ruminative coping_T2_	1.57	0.65	0.72	0.47	0.43*	(0.95)	0.81*	0.60*	−0.34*	−0.04
3. Confrontative coping_T2_	1.99	0.30	0.39	0.43	0.22*	0.39*	(0.76)	0.52*	−0.31*	−0.02
4. Workplace resource depletion_T3_	2.13	0.60	0.58	0.51	0.07	0.13*	0.08*	(0.87)	−0.61	0.00
5. Workplace goal attainment_T3_	3.64	0.59	0.58	0.50	−0.08*	−0.10*	−0.01	−0.33*	(0.91)	−0.02
6. Workplace affiliation disposition_T0_	3.29	0.90								(0.86)

N_Level-2_ = 214, N_Level-1_ = 1,611. M = composite mean of factor indicators. ICC = Intraclass correlation coefficient. We calculated the ICC as SD_Level-2_/(SD_Level-2_ + SD_Level-1_). Omega reliabilities are in parentheses on the diagonal. Level-2 correlations are above the diagonal. Level-1 correlations are below the diagonal. *p < 0.05.

We assessed the factor structure through confirmatory factor analyses (see Table 2). We employed both maximum likelihood and Bayesian estimators to verify the results. The hypothesized factor structure demonstrated a satisfactory level of fit. To confirm the discriminant validity of this structure, we also conducted CFAs for alternative models. The outcomes revealed that the alternative models exhibited a significantly poorer fit to the data than the hypothesized model.

**Table 2:** Confirmatory factor analyses models’ fit indices.

CFA models	χ^2^	*df*	∆ χ^2^(∆*df*)	CFI	TLI	RMSEA	SRMR_Level-1_	SRMR_Level-2_	AIC	BIC
Hypothesized model	560.712	249	*p* < 0.001	0.979	0.974	0.028	0.031	0.064	44,790.518	45,420.518
Alternative model 1^a^	8,487.574	277	*p* < 0.001	0.453	0.388	0.136	0.198	0.204	52,661.380	53,140.610
Alternative model 2^b^	1,232.438	258	*p* < 0.001	0.935	0.922	0.048	0.061	0.069	45,444.244	46,025.782
Alternative model 3^c^	4,308.056	271	*p* < 0.001	0.731	0.692	0.096	0.153	0.253	48,493.862	49,005.400

N_Level-2_ = 214; N_Level-1_ = 1,611. The statistical significance of the model comparison between alternative and hypothesized models is assessed by ∆*χ*^2^(∆*df*).

a All indicators load on same factor.

b Indicators of confrontation and rumination load on same factor.

c Indicators of workplace anger, ruminative coping, and confrontative coping load on same factor.

### Hypotheses testing

We present the results of our Bayesian structural equation modeling analyses for the direct and indirect effects in Tables 3, 4, respectively. Support was found for Hypothesis 1a, as workplace anger was positively associated with ruminative coping (E(*γ*) = 0.392, *p* < 0.001). Similarly, support was found for Hypothesis 1b, with workplace anger positively related to confrontative coping (E(*γ*) = 0.191, *p* < 0.001).

**Table 3:** Unstandardized coefficient estimates and posterior standard deviations of direct effects.

Variables	Ruminative coping_T2_		Confrontative coping_T2_		Workplace resource depletion_T3_		Workplace goal attainment_T3_	
	Estimate	*SD*	Estimate	*SD*	Estimate	*SD*	Estimate	*SD*
Level-2								
Workplace affiliation disposition_T0_	−0.045	0.046	−0.006	0.024	0.008	0.048	−0.017	0.048
Level-1								
Workplace anger_T1_	0.392*	0.080	0.191*	0.036	0.038	0.046	−0.074	0.042
Ruminative coping_T2_					0.108*	0.039	−0.070*	0.035
Confrontative coping_T2_					0.032	0.079	0.075	0.075
Workplace affiliation disposition_T0_								
× Workplace anger_T1_	−0.013	0.089	0.003	0.034				
× Ruminative coping_T2_					−0.136*	0.040	−0.021	0.036
× Confrontative coping_T2_					0.135	0.082	0.154*	0.078

N_Level-2_ = 214, and N_Level-1_ = 1,611. *p < 0.05.

**Table 4:** Unstandardized coefficient estimates and posterior standard deviations of indirect effects.

Indirect effects	Test of conditional effects		
	Estimate	CI LL	CI UL
Workplace anger → Ruminative coping → Workplace resource depletion	0.041*	0.012	0.079
Workplace anger → Confrontative coping → Workplace resource depletion	0.006	−0.024	0.039
Workplace anger → Ruminative coping → Workplace goal attainment	−0.026*	−0.060	−0.001
Workplace anger → Confrontative coping → Workplace goal attainment	0.014	−0.016	0.045

N_Level-2_ = 214, and N_Level-1_ = 1,611. Estimate = unstandardized parameter estimate of indirect effect. CI LL = lower limit of 95% credibility interval. CI UL = upper limit of 95% credibility interval. *p < 0.05.

In line with Hypothesis 2a, ruminative coping was found to be positively related to workplace resource depletion (E(*γ*) = 0.108, *p* = 0.004). However, contrary to Hypothesis 2b, confrontative coping showed no positive association with workplace resource depletion (E(*γ*) = 0.032, *p* = 0.674).

Support for Hypothesis 3a was found, indicating a negative relation between ruminative coping and workplace goal attainment (E(*γ*) = −0.070, *p* = 0.046). Hypothesis 3b was not supported, as confrontative coping did not show a positive relation with workplace goal attainment (E(*γ*) = 0.075, *p* = 0.288).

Support was found for Hypothesis 4a, revealing a positive relation between workplace anger and workplace resource depletion via ruminative coping (E(*indirect*) = 0.041, *p* = 0.004). Hypothesis 4b was not supported, as workplace anger was not positively related to workplace resource depletion via confrontative coping (E(*indirect*) = 0.006, *p* = 0.674).

Hypothesis 5a was supported, showing a negative relation between workplace anger and workplace goal attainment via ruminative coping (E(*indirect*) = −0.026, *p* = 0.046). Hypothesis 5b was not supported, as workplace anger was not positively related to workplace goal attainment via confrontative coping (E(*indirect*) = 0.014, *p* = 0.288).

### Exploratory analyses

**Figure 2:**
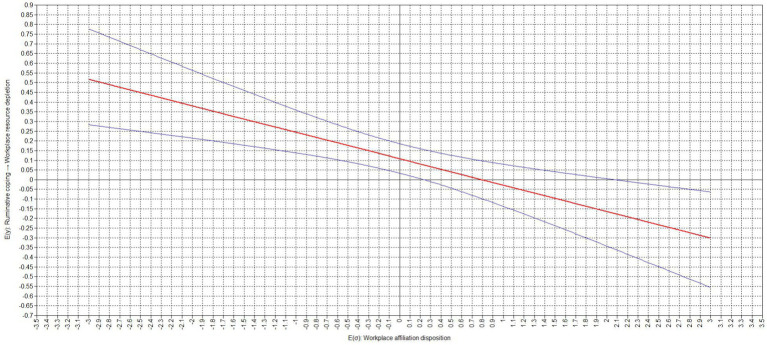
Region of significance for cross-level interaction effect of workplace affiliation disposition on the relation between ruminative coping and workplace resource depletion.

**Figure 3:**
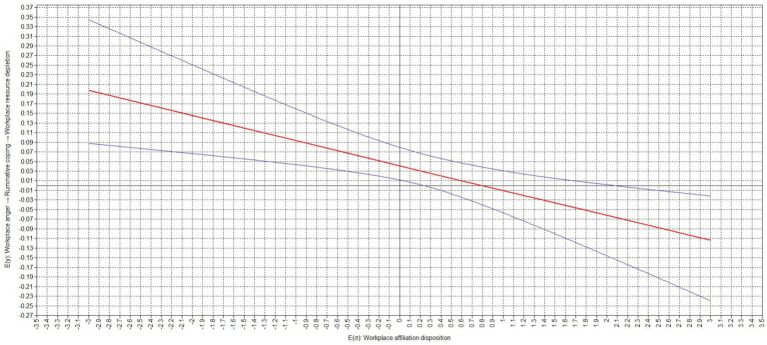
Region of significance for cross-level interaction effect of workplace affiliation disposition on the relation workplace anger and workplace resource depletion via ruminative coping.

Table 4 presents the results of our analyses on the conditional effects. Our study found that the relation between ruminative coping and workplace resource depletion was moderated by an employee’s workplace affiliation disposition (moderation effect = −0.136, *p* < 0.001). Our results indicate that the relation between ruminative coping and workplace resource depletion was significant for low (−1 standard deviation; simple slope = 0.245, *p* < 0.001) and but not high (+1 standard deviation; simple slope = −0.029, *p* = 0.590) expressions of an employee’s workplace affiliation disposition. The difference between the slopes was significant (difference = −0.273, *p* < 0.001). Similarly, the relation between workplace anger and workplace resource depletion via ruminative coping was significant for low (−1 standard deviation; conditional effect = 0.093, *p* < 0.001) but not high (+1 standard deviation; conditional effect = −0.011, *p* = 0.590) expressions of an employee’s workplace affiliation disposition. The difference between the slopes was significant (difference = −0.104, *p* < 0.001). The corresponding region of significance plots, presented in Figures 2, 3, show that the relation between ruminative coping and workplace resource depletion as well as the relation between workplace anger and workplace resource depletion via ruminative coping did become non-significant with values of a workplace affiliation disposition of equal to or greater than 0.240 standard deviations above the sample mean, while becoming negative and significant with values of a workplace affiliation disposition of equal to or greater than 2.080 standard deviations above the sample mean (Table 5).

**Table 5:** Unstandardized coefficient estimates and credibility intervals of conditional effects.

Effects	Test of conditional effects		
	Estimate	CI LL	CI UL
Ruminative coping → Workplace resource depletion			
Unconditional direct effect	0.108*	0.033	0.185
Conditional direct effect upon low (−1 SDs) workplace affiliation disposition	0.245*	0.138	0.359
Conditional direct effect upon high (+1 SDs) workplace affiliation disposition	−0.029	−0.138	0.079
Difference of high and low workplace affiliation disposition	−0.273*	−0.437	−0.123
Workplace anger → Ruminative coping → Workplace resource depletion			
Unconditional indirect effect	0.041*	0.012	0.079
Conditional indirect effect upon low (−1 SDs) workplace affiliation disposition	0.093*	0.043	0.159
Conditional indirect effect upon high (+1 SDs) workplace affiliation disposition	−0.011	−0.057	0.031
Difference of high and low workplace affiliation disposition	−0.104*	−0.192	−0.040
Confrontative coping → Workplace goal attainment			
Unconditional direct effect	0.075	−0.069	0.227
Conditional direct effect upon low (−1 SDs) workplace affiliation disposition	−0.077	−0.293	0.129
Conditional direct effect upon high (+1 SDs) workplace affiliation disposition	0.229*	0.022	0.458
Difference of high and low workplace affiliation disposition	0.308*	0.010	0.631
Workplace anger → Confrontative coping → Workplace goal attainment			
Unconditional indirect effect	0.014	−0.016	0.045
Conditional indirect effect upon low (−1 SDs) workplace affiliation disposition	−0.014	−0.056	0.025
Conditional indirect effect upon high (+1 SDs) workplace affiliation disposition	0.043*	0.004	0.091
Difference of high and low workplace affiliation disposition	0.058*	0.002	0.125

N_Level-2_ = 214, and N_Level-1_ = 1,611. Estimate = unstandardized parameter estimate of indirect effect. CI LL = lower limit of 95% credibility interval. CI UL = upper limit of 95% credibility interval. SDs = standard deviations. *p < 0.05.

**Figure 4:**
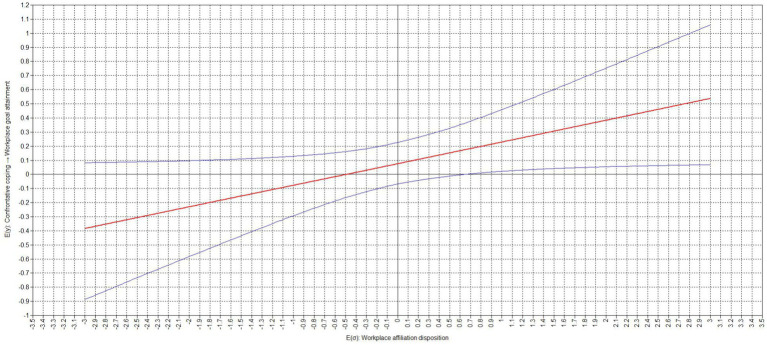
Region of significance for cross-level interaction effect of workplace affiliation disposition on the relation between confrontative coping and workplace goal attainment.

**Figure 5:**
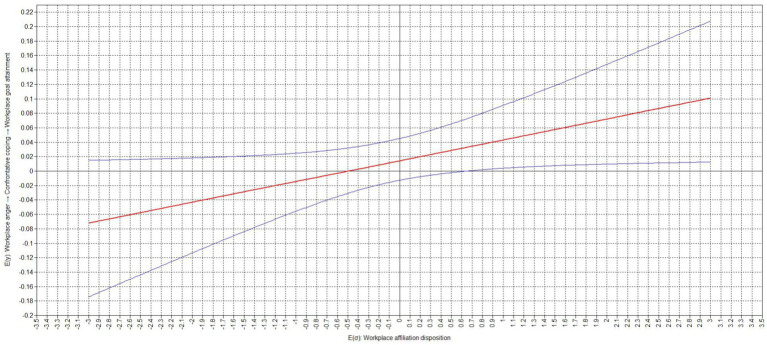
Region of significance for cross-level interaction effect of workplace affiliation disposition on the relation workplace anger and workplace goal attainment via confrontative coping.

Our results also showed that the relation between confrontative coping and workplace goal attainment was moderated by an employee’s workplace affiliation disposition (moderation effect = 0.154, *p* = 0.042). Our results indicate that the relation between confrontative coping and workplace goal attainment was still insignificant for low (−1 standard deviation; simple slope = −0.077, *p* = 0.452) but not high (+1 standard deviation; simple slope = 0.229, *p* = 0.032) expressions of an employee’s workplace affiliation disposition. The difference between the slopes was significant (difference = 0.308, *p* = 0.042). Similarly, the relation between workplace anger and workplace goal attainment via confrontative coping was also still insignificant for low (−1 standard deviation; conditional effect = −0.014, *p* = 0.452) but not high (+1 standard deviation; conditional effect = 0.043, *p* = 0.032) expressions of an employee’s workplace affiliation disposition. The difference between the slopes was significant (difference = 0.058, *p* = 0.042). The corresponding region of significance plots, presented in Figures 4, 5, show that the relation between confrontative coping and workplace goal attainment as well as the relation between workplace anger and workplace goal attainment via confrontative coping did not become negative and significant, while becoming positive and significant with values of a workplace affiliation disposition of equal to or greater than 0.640 standard deviations above the sample mean.

## Discussion

In this manuscript, our primary focus was on examining the intricate relation between workplace anger, workplace resource depletion, and goal attainment while taking into account the impact of coping strategies and individual differences. Our main objective was to challenge the common belief that workplace anger always impedes work outcomes. To achieve this goal, we developed a theoretical model based on affective events theory (Weiss and Cropanzano, 1996) and insights from cognitive theories of emotions (Lazarus, 1991; Frijda, 1987; Moors et al., 2013). Our conceptual model suggests that workplace anger may be positively related to workplace resource depletion and may have negative associations with workplace goal attainment in certain situations, but could also have a positive relation with workplace goal attainment under other circumstances. Factors such as coping strategies for managing anger and individual differences among employees were taken into consideration in this model. In order to test our theoretical framework, we gathered data from a group of full-time employees across various industries using a daily time-lagged experience-sampling method over a two-week period. Contrary to previous research, our results indicated a lack of significant association between workplace anger and workplace resource depletion, as well as a positive connection between workplace anger and goal attainment, depending on the coping mechanisms employed by employees when faced with anger-triggering situations, as well as their workplace affiliations.

### Theoretical implications

Our results have significant theoretical implications. Firstly, our findings suggest that the assumptions of affective events theory (Weiss and Cropanzano, 1996) and cognitivist accounts of emotion, which propose an adverse relation between workplace anger and resource depletion, appear to be supported by empirical evidence, although only to a limited extent. The effect size of the relation between workplace anger and resource depletion is below the threshold for a small effect (E(*β*) = 0.041, *r* = 0.091; Cohen, 1988; Gignac and Szodorai, 2016), leading us to question the practical validity of these assumptions. Additionally, as this relation was only significant when mediated by coping strategies, it is important to consider that the interplay between affective responses and work outcomes is contingent upon the intersection of these coping strategies. Therefore, it may be beneficial to integrate coping strategies into affective events theory (Weiss and Cropanzano, 1996) to provide a more comprehensive understanding of the relation between emotions and work outcomes.

The importance of integrating coping strategies into affective events theory (Weiss and Cropanzano, 1996) is further clarified when considering the various intersecting effects of coping strategies in the relation between workplace anger and work outcomes. Our research supports the concept of cognitivist accounts of emotions (Frijda, 1987; Lazarus, 1991; Moors et al., 2013) by demonstrating how various coping strategies intersect and direct the valence of the relation between workplace anger and work outcomes in different ways. Specifically, we found that the connection between workplace anger and goal attainment is significantly adverse when ruminative coping is considered but becomes negligible when confrontative coping is taken into account. This same pattern is also evident in the link between workplace anger and resource depletion. Notably, the indirect effects of workplace anger on work outcomes through confrontative coping, as well as the direct effects between confrontative coping and work outcomes, are close to zero. This suggests that the lack of significant results may not be due to a lack of statistical power, but rather indicate a practical buffering effect of confrontative coping between workplace anger and work outcomes. This highlights the interplay between coping strategies and these constructs, emphasizing the crucial role of coping strategies in the relation between workplace anger and work outcomes. Therefore, incorporating coping strategies into affective events theory (Weiss and Cropanzano, 1996) could improve our understanding of how workplace anger relates to work outcomes.

However, we also noted that the relations between workplace anger and work outcomes depended on how an employee expressed their disposition toward workplace affiliation. Prior research has not thoroughly explored individual employee-level factors within the context of affective events theory, making these findings especially significant. This disposition appears to play a crucial role in shaping the relations between workplace anger, coping strategies, and work outcomes. It is important to note that the relation between workplace anger and resource depletion through ruminative coping became insignificant when considering the interaction between a high disposition toward workplace affiliation and these coping strategies. Conversely, the relation between workplace anger and goal attainment through confrontative coping became positive and significant in the same context, aligning with principles of cognitivist theories of emotions (Lazarus, 1991). This highlights the potential value of integrating this interaction into affective events theory (Weiss and Cropanzano, 1996), in conjunction with other theoretical propositions of constructivist accounts of emotions (Lazarus, 1991; Frijda, 1987; Moors et al., 2013), to enhance our comprehension of how affective responses are linked to work outcomes.

### Practical implications

Our study suggests that leaders may benefit from adopting a dual approach when dealing with elevated levels of anger among their employees. The first aspect of this strategy involves encouraging confrontational behavior among employees without imposing penalties. This can be achieved through providing assertiveness training to help employees confront others at work (see Abdelaziz et al., 2020; Omura et al., 2017; Speed et al., 2018).

Simultaneously, it is crucial to implement interventions that enhance workplace affiliation. Improving the quality of relations among employees by fostering workplace friendships (Methot et al., 2016; van Dick et al., 2004) or creating mutually dependent work teams (Dietz and Fasbender, 2021) should help increase the desire of employees to affiliate with one another, at least in the short term.

Our research findings suggest that the positive relation between anger and goal attainment is dependent on the interplay of assertiveness and affiliation factors. Thus, solely focusing on one type of intervention may not be as effective. Instead, implementing both assertiveness and affiliation strategies concurrently can lead to higher employee goal attainment rates, ultimately improving organizational performance on a daily basis.

### Limitations

In interpreting the results of our study, it is imperative to consider its limitations. First, self-report measures may have introduced common method bias (Doty and Glick, 1998), which could compromise the validity of our findings. Therefore, it is advisable for forthcoming research efforts to integrate other-report study design components, such as those that evaluate employee dyads. This approach can help mitigate the potential bias through inter-method reliability.

Second, our time-based sampling strategy may have impacted our estimates by missing event-based variance due to fixed sampling intervals (Trull and Ebner-Priemer, 2009). We attempted to address this limitation by setting narrow sampling intervals. Nonetheless, future research could consider incorporating random survey prompts to reduce this potential bias further.

Our study did not examine the precise sources and targets of workplace anger (organizational, non-organizational, metaphysical) or the employee’s situational control related to such workplace anger (Lazarus, 1991; Lazarus and Folkman, 1984; Potegal et al., 2010). This complexity was beyond the scope of our study design. Although our theoretical framework and results should still hold under these distinctions, we recommend that future researchers incorporate the sources and targets of workplace anger as well as situational control into their study designs to enhance understanding further about the mechanisms underlying our conceptual model (see also Schwarzmüller et al., 2018, for a good starting point for this direction).

Overall, it is important to note that these limitations may somewhat restrict the generalizability and viability of our interpretations and results. Therefore, future research is encouraged to address these challenges through replication and conceptual expansion.

### Future research directions

One potential direction for future research is to experimentally replicate the relations that were proposed and tested in the current paper. Our approach lacks internal validity, indicating that conducting experimental studies could help address this limitation. Vignette studies could be used by future researchers to test our assumptions and bring both internal and external validity to the assumed relations (Aguinis and Bradley, 2014).

Additionally, it may be beneficial for future research to investigate the reasons behind the impact of a workplace affiliation disposition on the relation between ruminative coping and workplace resource depletion as well as the relation between confrontative coping and workplace goal attainment. Qualitative research, such as in-depth interviews, could be utilized to uncover the underlying reasons for these associations (see Fitness, 2000). Following this, conceptual research could identify commonalities from the interviews (Locke, 2007), which could then be tested in experience-sampling studies and replicated across various business settings (Gabriel et al., 2019).

Future research should further explore the lack of a significant effect of confrontative coping on resource depletion. It is worth investigating whether organizational and leadership practices that incorporate confrontative approaches toward anger-inducing employees could provide a viable framework for sustaining productivity while ensuring both short-and long-term employee well-being. Additionally, future studies could examine whether these effects—particularly those related to different expressions of workplace affiliation—remain consistent across various workplace cultures and organizational roles.

## Conclusion

The primary objective of organizations is to be resourceful and achieve their goals (Mohr, 1973; Sekaran and Snodgrass, 1989). While anger has traditionally been viewed as a barrier to these objectives (see Jäger et al., 2017; Wong et al., 2017; but see also Schmitt et al., 2019), our study challenges this notion. We have found evidence suggesting that anger is not linked to resource depletion, and in fact, it can be advantageous for employees in reaching their work-related goals, especially when the individual experiencing anger is integrated within the organizational framework and well-established within it.

## Data Availability

The raw data supporting the conclusions of this article will be made available by the authors without undue reservation.

## Footnotes

^1^*ν*, *λ*, *β*, *α* ~ N(0, ∞); *θ* ~ IG(−1, 0); *ψ* ~ IW(0, −p-1) (see Muthén and Muthén, 2017, p. 775, for notation).

## Author’s note

Data and materials are shared openly as part of the publication of this article at https://osf.io/3tdwv/?view_only=30658bc72bc743e28775dd6876cce1d5.
